# Reproductive factors and molecular subtypes of breast cancer among premenopausal women in Latin America: the PRECAMA study

**DOI:** 10.1038/s41598-018-31393-7

**Published:** 2018-08-30

**Authors:** Isabelle Romieu, Carine Biessy, Marion Carayol, Mathilde His, Gabriela Torres-Mejía, Angélica Ángeles-Llerenas, Gloria Inés Sánchez, Roberto Jaramillo, Edgar Navarro, Carolina Porras, Rebecca Ocampo, Ana Cecilia Rodriguez, Maria Luisa Garmendia, Eva Bustamante, Magali Olivier, Peggy Porter, Sabina Rinaldi, On behalf of the PRECAMA team, Jenny Tejeda, Fancy Gaete, Jose Soto, Gonzalo Alberto Angel, Carlos Andres Ossa, William H. Arias, Gabriel Bedoya, Mauricio Borrero, Alicia Cock-Rada, Israel Díaz-Yunez, Carolina Echeverri, Fernando Herazo, Angel Hernández, Yorlany Rodas Cortes, Bernal Cortes, Paula Gonzalez, Diego Guillen

**Affiliations:** 1 Section of Nutrition and Metabolism, International Agency for Research on Cancer, Lyon, France; 2 Val D’Aurelle Montpellier Cancer Institute (ICM), Montpellier, France; 3 Centre for Population Health Research, National Institute of Public Health, Cuernavaca, Mexico; 4 Group Infection and Cancer, School of Medicine, University of Antioquia, Medellín, Colombia; 5 Hemato Oncologos, Cali, Colombia; 6 Grupo Proyecto UNI-Barranquilla, Universidad del Norte, Barranquilla, Colombia; 7 Agencia Costarricense de Investigaciones Biomédicas (ACIB)-Fundación INCIENSA, San José, Costa Rica; 8 Instituto de Nutrición y de Tecnología de los Alimentos, Santiago, Chile; 9 Instituto Oncológico Fundación Arturo López Pérez, Providencia, Santiago, Chile; 10 Molecular Mechanisms and Biomarkers Group, International Agency for Research on Cancer, Lyon, France; 11 Divisions of Human Biology and Public Health Sciences, Fred Hutchinson Cancer Research Center, Seattle, USA; 12 Hospital Santiago Oriente Dr. Luis Tisné Brousse, Santiago, Chile; 13 National Institute of Cancer, Santiago, Chile; 14 Torre Médica Las Américas, Medellín, Colombia; 15 GENMOL Group, Natural and Basic Sciences Faculty, University of Antioquia, Medellín, Colombia; 16 Clínica Vida Fundacion, Medellín, Colombia; 17 Instituto de Cancerología Las Américas, Medellín, Colombia; 18 Imágenes Diagnósticas y Biotecnología Reproductiva, Cediul S.A., Barranquilla, Colombia; 19 Clínica Bonnadona Prevenir, Barranquilla, Colombia; 20 Torre Médica El Tesoro, Medellín, Colombia

## Abstract

Etiological differences among breast cancer (BC) subtypes have not been clearly established, especially among young women in Latin America. This study examined the relationship between reproductive factors and BC subtypes among 288 BC cases (20–45 years) and population-based matched controls in four Latin American countries. Immunohistochemistry was determined centrally. Associations between BC and reproductive factors were determined. Older age at first full-term pregnancy (FFTP) (Odds Ratio (OR) = 1.11; 95% Confidence Interval (CI), 1.04–1.19 per year), longer time between menarche and FFTP (OR = 1.12; 95%CI: 1.04–1.20 per year), and older age at last pregnancy (OR = 1.10; 95%CI, 1.02–1.19 per year) were associated with an increased risk of estrogen receptor positive (ER+) tumors (n = 122). Ever pregnant (OR = 0.35; 95%CI, 0.13–0.96), number of childbirths (OR = 0.64; 95%CI, 0.47–0.87 per child), time since last birth (OR = 0.92; 95%CI, 0.85–0.99 per year), and history of breastfeeding (OR = 0.23; 95%CI, 0.09–0.58) were inversely associated with the risk of ER+ tumor. Older age at menarche (OR = 0.63; 95%CI, 0.45–0.89 per year) and longer duration of breastfeeding (OR = 0.97; 95%CI, 0.94–1.01 per month) were inversely associated with estrogen receptor negative (ER-) tumors (n = 48). Reproductive factors may be differentially associated with BC subtypes in young Latin American women.

## Introduction

Breast cancer (BC) in young women is a leading cause of death and remains so despite more intensive treatment than in older women^1^. This may be related to different risk factors and tumor biology in young women. Receptor expression and tumor genomics may be associated with distinct reproductive, personal, and behavioral factors (e.g. obesity and diet). In Latin American (LA) women, 20% of BC occurs at ages 20–45 years, a higher proportion than in Westernized populations^2^, and this substantial number of incident BC cases among young LA women can only be partly explained by the population age structure^3^. This understudied population is currently undergoing significant reproductive and lifestyle transitions^4^ that offer unique contrasts in exposures to study factors associated with BC subtypes.

Reproductive factors are the group of factors with the strongest and most consistent associations with BC risk^5^. Known risk factors associated with BC include age at menarche, age at first birth, and parity. Increasing parity is protective against breast cancer, but the effects of younger age at first birth and parity appear to be smaller in women diagnosed with BC at age <40 years, and there is a transient increase in the risk of BC after each birth^6,7^. Few studies have focused on risk factors for BC in young women, and these studies have mainly been conducted in Caucasian women^8^, with only limited data in Latin American or U.S Hispanic women^2,9^. In addition, in-depth knowledge of molecular and pathological characteristics of BC in young women is lacking, in particular in Latin America, with major consequences for cancer treatment and survival. We therefore conducted a multicenter population-based case–control study on BC in women aged 20 to 45 years across four Latin American countries (Chile, Colombia, Costa Rica, and Mexico) to determine BC subtypes and associated risk factors. In this report, we focus on the role of reproductive factors.

## Methods

### PRECAMA study

The PRECAMA study is an ongoing population-based case-control study coordinated by the International Agency for Research on Cancer (IARC) and involves scientific teams in four LA countries (Chile, Colombia, Costa Rica, and Mexico). Through IARC and a central laboratory (the Porter Lab) at the Fred Hutchinson Cancer Research Center (FHCRC) in Seattle, the research teams have harmonized and standardized protocols in the participating centers for recruitment of cases and controls, collection and storage of blood samples, tumor block fixation and handling, and pathology review. A central web-based database has been developed. The pilot phase of the study started in 2012. All participants gave written informed consent before enrolment, and the study protocols were approved by the institutional review boards of Chile (Oncologic Institute Foundation Arturo Lopez Pérez and National Cancer Institute), Colombia (Cancer Institute Las Americas and University of Antioquia), Costa Rica (Costa Rican Institute of Clinical Research (ICIC) and Center for Strategic Development and Information in Health and Social Security (CENDEISSS) of the Costa Rican Social Security Fund (CCSS)), Mexico (National Institute of Public Health and the Social Security Mexican Institute), and the International Agency for Research on Cancer (IARC). All methods were performed in accordance with the relevant guidelines and regulations of these approvals.

### Selection of cases

Incident cases of primary invasive BC were recruited from large general or cancer-specific hospitals or private oncology institutes providing services to large urban populations with a wide range of socioeconomic status. The inclusion criteria were: age 20 to 45 years, residence for at least 3 years in the same city district and having an incident primary invasive BC with positive biopsy and clinical staging according to tumor–node–metastasis (TNM) standards. Women with a recent biopsy diagnosis of BC (incident case) or a referral to the breast surgeon because of suspicious mammography that was confirmed as BC were approached by a nurse and/or a breast surgeon and invited to participate in the study before starting any treatment.

### Selection of controls

Controls were randomly selected from the underlying population residing in the same city district (neighborhood) as the case for at least 3 years using a standardized multilevel sampling frame. In Chile, because of logistic constraints, some controls were referred by the cases (acquaintances but not relatives). Controls were matched to cases on age (±3 years), city district of residence, and health insurance institution.

### Exposure assessment

During the recruitment visit of cases and controls, trained nurses administered the lifestyle and food frequency questionnaires, conducted anthropometric measurements (weight, height, waist circumference, hip circumference, and sitting height), and collected fasting biological samples (blood and spot urine samples).

The lifestyle questionnaire included information on socioeconomic status during infancy and early childhood (based on parental education and occupation, place of residence, and type of housing during childhood), health and reproductive history (age at menarche, pregnancy, number of births, age at each birth, breastfeeding, history of benign breast disease, use of hormones (e.g. oral contraceptives), smoking habits, alcohol intake, maximum attained weight, body silhouette at different ages, physical activity (household and recreational) and hours per day spent sedentary (e.g. watching television), and family history of cancer.

### Pathology review and immunohistochemical analyses

Prepared histology sections from tumor biopsy, obtained before any treatment, or obtained during surgery if no adjuvant therapy was administered, were reviewed for diagnosis, tumor grade, lymphovascular invasion, and stromal and lymphocyte response in a centralized laboratory in Seattle, USA (the Porter Lab, FHCRC). Tumor samples with ≥1% immunostained tumor cell nuclei were considered positive (Estrogen receptor positive (ER+), Progesterone receptor positive (PR+)). For human epidermal growth factor receptor 2 (HER2), samples were considered positive if there was strong membrane immunostaining (3+) and negative otherwise. In addition, the expression percentages of p53 (classified as positive if >10%) and the proliferation marker Ki-67 (classified as high if >14%) were determined. Triple-negative (TN) tumors were defined as estrogen receptor negative (ER−), progesterone receptor negative (PR−), and human epidermal growth factor receptor 2 negative (HER2−), and among the TN tumors basal-like cancers were defined as ER−, PR−, HER2−, and Epidermal growth factor receptor positive *(*EGFR+) and/or cytokeratin 5/6 positive (CK5/6+).

### Statistical analyses

Descriptive statistics (median, 10th to 90th percentiles, and proportions) were calculated overall and stratified by tumor subtypes, considering ER+, ER−, and TN tumor classification. For categorical variables cut-off points were based on the distribution among controls. Conditional logistic regression was used to calculate odds ratio (OR) and 95% confidence interval (CI) for the association of reproductive factors. In addition to age at menarche, ever pregnant (full term pregnancy), age at first and last full term pregnancy, time between menarche and first full term pregnancy, number of childbirths, history of breastfeeding and duration of breastfeeding, we created variables by combining number of childbirths and breastfeeding (categorical), number of childbirths and age at first full-term pregnancy (FFTP), using 21 years (the median age at FFTP) as the cut-off (categorical), and number of childbirths and time since last birth (categorical). Stratified analyses were conducted on ER+ and ER− tumors and TN tumors and compared with their corresponding matched controls. To determine the effect of each reproductive factor, our models were adjusted for age at first birth, number of childbirths, duration of breastfeeding, education level, physical activity, waist circumference, family history of breast cancer and history of benign breast disease. Our final multivariate models, adjusted for factors that changed the risk estimate by more than 10%, included maternal education level (low, medium, or high), physical activity (moderate physical activity in hours per day), waist circumference (continuous), and history of benign breast disease (yes/no). In reporting the results, a two-sided *P* value less than 0.05 was considered statistically significant. Non-linear association between breastfeeding duration and BC was explored using splines with knots at 6 and 12 months^10^. Analyses were conducted using SAS (version 9.4, SAS Institute, Cary, NC) and STATA (version 14) software

## Results

### Characteristics of the study population

Among 288 matched cases and controls, the median age at recruitment was 40 years. Compared with controls, cases were more educated (34% vs 16% with a high level) and had a higher prevalence of history of benign breast disease (35% vs 13%). Among reproductive variables, compared with controls, cases were less likely to ever being pregnant (83% vs 92%), and, when they had a child, had fewer children (19% vs 32% had ≥3 children), and were older at first (42% vs 24% for ≥25 years) and last pregnancy (48% vs 38% for >30 years). Compared to controls, cases had longer time between menarche and FFTP (63% vs 44% for time >8 years), shorter time since last birth (42% vs 54% for time ≥10 years), and were less likely to have breastfed (89% vs 95%), and when they breastfed, they did so for a shorter duration (40% vs 60% for duration >12 months). Body mass index was slightly higher in controls than in cases (28.3 kg/m^2^ vs 26.1 kg/m^2^), as was median waist circumference (93 cm vs 90 cm). Moderate physical activity was higher in controls (2.7 vs 1.5 hours per day), while sedentary behavior was higher in cases than in controls (14.3 vs 12.9 hours per day) (Table 1).

**Table 1:** Characteristics of the study population for cases and controls.

Characteristic^a,b^	Controls (*n* = 288)	Cases (*n* = 288)
**Age at recruitment (years)**	40 (31–44)	40 (30–44)
**Education level**		
≤Primary school	82 (28%)	50 (17%)
Secondary school	160 (56%)	142 (49%)
>Secondary school	46 (16%)	96 (34%)
**Family history of breast cancer**	15 (5%)	17 (6%)
**History of benign breast disease**	37 (13%)	101 (35%)
**Age at menarche (years)**	12 (11–15)	12 (10–15)
≤12	156 (54.2%)	154 (53.5%)
>12	132 (45.8%)	134 (46.5%)
**Pregnancy ever**	266 (92%)	240 (83%)
**Age at FFTP (years)** ^**c**^	20 (16–29)	23 (17–32)
<20	107 (40%)	66 (28%)
20–24	95 (36%)	71 (30%)
≥25	64 (24%)	101 (42%)
**Time between menarche and FFTP (years)** ^**c**^	8 (3–16)	11 (4–20)
≤8	150 (56%)	87 (37%)
>8	116 (44%)	151 (63%)
**Age at last pregnancy (years)** ^**c**^	28 (22–35)	30 (23–38)
≤25	93 (35%)	63 (26%)
26–30	73 (27%)	62 (26%)
>30	100 (38%)	113 (48%)
**Time since last birth (years)** ^**c**^	10 (3–18)	8.5 (2–18)
<10	123 (46%)	138 (58%)
≥10	143 (54%)	100 (42%)
**Parity** ^**d**^	2 (1–3)	2 (0–3)
Nulliparous	22 (8%)	48 (17%)
1 child	52 (18%)	84 (30%)
2 children	121 (42%)	96 (34%)
≥3 children	92 (32%)	53 (19%)
**Breastfeeding ever** ^**c**^	252 (95%)	213 (89%)
**Duration of breastfeeding (months)** ^**c**^	20 (4–48)	12 (2–42)
Never	14 (5%)	27 (11%)
≤12	92 (35%) → (37%)	117 (49%) → (55%)
>12	160 (60%) → (63%)	96 (40%) → (45%)
**Anthropometric measurements**		
Weight (kg)	68.5 (53–92)	64.5 (51–81)
Height (m)	1.56 (1.50–1.65)	1.57 (1.49–1.66)
Body mass index (kg/m^2^)	28.3 (21.8–36.4)	26.1 (20.7–32.9)
Waist circumference (cm)	93 (76–112)	90 (75–106)
**Physical activity (hours per day)**		
Moderate physical activity	2.7 (0.7–7.0)	1.5 (0.2–5.4)
Light physical activity/Sedentary	12.9 (8.4–16.1)	14.3 (8–17)

^a^Median (10th percentile to 90th percentile) or number (%).

^b^Missing cases/controls: 8 missing (7/1) for parity (number of children); 2 missing (2/0) for age at FFTP, age at last pregnancy, time since last birth, and time between menarche and FFTP; 3 missing (1/2) for height, waist circumference, and body mass index.

^c^Parous women only; FFTP: first full-term pregnancy.

^d^Parity: number of childbirths.

Table 2 describes BC subtypes according to standardized immunohistochemistry (IHC) for the 169 cases for which IHC is currently available. Of these cases, 72% were ER+, 28% were ER−, 19% were HER2+, and 21% were TN (ER−/PR−/HER2−). Among the TN, 92% (33/36) were classified as basal-like. The proportions of p53 positive (>10%) and Ki-67 high (>14%)^11^ were the largest in TN tumors.

**Table 2:** Description of breast cancer cases (n = 170) according to hormone receptor status and other tumor characteristics.

Hormone receptor status^a^	All cases Col (%)	P53 positive^b^ Row (%)	Ki-67 positive^c^ Row (%)	Age (years)^d^
ER+	122 (72%)	20 (16%)	88 (72%)	40.5 (30–45)
ER−	48 (28%)	24 (51%)	45 (96%)	38.5 (29–44)
PR+	123 (72%)	21 (17%)	88 (72%)	41.0 (30–45)
PR−	47(28%)	24 (51%)	45 (96%)	38 (29–44)
HER2+	32 (19%)	16 (50%)	28 (88%)	39.5 (28–44)
HER2−	138 (81%)	29 (21%)	105 (76%)	40 (30–45)
Triple-negative (ER−/PR−/HER2−)	36 (21%)	19 (53%)	35 (97%)	38 (30–45)
*Of which Basal-like* (*TN* + *EGFR*+ *and/or CK5/6*+)	*33* (*20%*)	*17* (*51%*)	*32* (*97%*)	*36* (*30–45*)

^a^ER: estrogen receptor; PR: progesterone receptor; HER2: human epidermal growth factor receptor 2. Overall score of ≥1% immunostained tumor cell nuclei was considered positive for ER+ or PR+; for HER2, was considered positive if membrane immunostaining 3+ and negative otherwise.

^b^p53 positive when >10%; 1 participant with missing data.

^c^Ki-67 high when >14%.

^d^Median (10th percentile to 90th percentile); 1 participant with missing data.

### Reproductive factors and overall breast cancer risk

In multivariate conditional logistic regression analyses (adjusting for education level, history of benign breast disease, waist circumference, and physical activity), older age at FFTP (OR = 1.06; 95% CI, 1.01–1.11 per year), longer time between menarche and FFTP (OR = 1.67; 95% CI, 1.06–2.64 comparing ≤8 years vs >8 years), and older age at last pregnancy (OR = 1.99; 95% CI, 1.09–3.61 comparing ≤25 years vs >30 years) were associated with an increased risk of BC. Older age at menarche (OR = 0.89; 95% CI, 0.80–0.99 per year), ever being pregnant (OR = 0.53; 95% CI, 0.29–0.97), a longer time since last birth (OR = 0.46; 95% CI, 0.28–0.77 comparing <10 years vs ≥10 years), higher number of childbirths (OR = 0.82; 95% CI, 0.67–1.00 per child), history of breastfeeding (OR = 0.56; 95% CI, 0.34–0.93), and a longer duration of breastfeeding among parous women (OR = 0.42; 95% CI, 0.18–0.94 comparing ≤12 months vs>12 months) were associated with a decreased risk of BC (Table 3).

**Table 3:** Odds ratios and 95% confidence intervals of association between breast cancer and reproductive factors.

Factor	N	Matched^a^	p-value^a^	Multivariate^b^	p-value^b^
	Cases/Controls	OR (95% CI)^c^		OR (95% CI)^c^	
**Education.level**					
≤Primary school	50/82	1.00		1.00	
Secondary school	142/160	1.87 (1.10–3.18)	**0.02**	1.78 (1.00–3.17)	**0.05**
>Secondary school	96/46	4.83 (2.60–8.98)	**<0.0001**	4.25 (2.19–8.27)	**<0.0001**
**Family history of breast cancer**	17/15	1.14 (0.56–2.34)	0.72	1.03 (0.46–2.34)	0.94
**History of benign breast disease**	101/37	3.46 (2.24–5.36)	**<0.0001**	3.43 (2.16–5.45)	**<0.0001**
***Reproductive variables***					
**Age at menarche (years)**	288/288	0.93 (0.85–1.01)	0.10	0.89 (0.80–0.99)	**0.04**
≤12	154/156	1.00		1.00	
>12	134/132	1.03 (0.74–1.43)	0.87	0.88 (0.60–1.30)	0.53
**Pregnancy ever**	240/266	0.41 (0.24–0.71)	**0.001**	0.53 (0.29–0.97)	**0.04**
**Age at FFTP (years)** ^**d**^		1.08 (1.04–1.13)	**<0.0001**	1.06 (1.01–1.11)	**0.01**
<20	66/107	1.00		1.00	
20–24	71/95	1.03 (0.62–1.69)	0.92	0.74 (0.41–1.33)	0.32
≥25	101/64	2.44 (1.47–4.04)	**0.001**	1.53 (0.82–2.83)	0.18
**Time between menarche and FFTP (years)** ^**d**^		1.09 (1.05–1.13)	**<0.0001**	1.07 (1.03–1.12)	**0.001**
≤8	87/150	1.00		1.00	
>8	151/116	1.98 (1.35–2.89)	**<0.0001**	1.67 (1.06–2.64)	**0.03**
**Age at last pregnancy (years)** ^**d**^		1.06 (1.02–1.10)	**0.004**	1.07 (1.03–1.13)	**0.003**
≤25	63/93	1.00		1.00	
26–30	62/73	1.25 (1.76–2.06)	0.38	1.31 (0.72–2.39)	0.37
>30	113/100	1.78 (1.07–2.95)	**0.03**	1.99 (1.09–3.61)	**0.02**
**Time since last birth (years)** ^**d**^		0.95 (0.91–0.99)	**0.009**	0.94 (0.90–0.99)	**0.01**
<10	138/123	1.00		1.00	
≥10	100/143	0.53 (0.34–0.81)	**0.004**	0.46 (0.28–0.77)	**0.003**
**Parity** ^d^ **(per child)**		0.66 (0.55–0.78)	**<0.0001**	0.82 (0.67–1.00)	**0.05**
Nulliparous	48/22	1.00		1.00	
1 child	84/52	0.76 (0.40–1.44)	0.40	0.89 (0.44–1.83)	0.76
2 children	96/121	0.36 (0.20–0.65)	**0.001**	0.53 (0.27–1.03)	0.06
≥3 children	53/92	0.25 (0.13–0.48)	**<0.0001**	0.51 (0.24–1.05)	0.07
**Breastfeeding ever**	213/252	0.43 (0.28–0.66)	**<0.0001**	0.56 (0.34–0.93)	**0.03**
**Duration of breastfeeding (months)** ^**d**^		0.98 (0.97–0.99)	**0.003**	0.99 (0.98–1.00)	0.08
Never	27/14	1.00		1.00	
≤12	117/92	0.80 (0.39–1.63)	0.53	0.73 (0.33–1.63)	0.45
>12	96/160	0.32 (0.15–0.66)	**0.002**	0.42 (0.18–0.94)	**0.04**

^a^Conditional logistic regression matched on age, country, city district, and health insurance institution (when appropriate).

^b^Conditional logistic regression matched on age, country, city district, and health insurance institution and adjusted for education level, history of benign breast disease, physical activity, and waist circumference when appropriate.

^c^ORs are given for both continuous (unit of change) and categorical variables.

^d^Parous women only; FFTP: first full-term pregnancy.

When analyses were stratified by history of breastfeeding (ever vs never), women who had ≥2 children and who had ever breastfed had a 48% lower risk of BC than nulliparous women (OR = 0.52; 95% CI, 0.27–0.98), while no association was observed among women who had one or more children and who had never breastfed (OR = 1.14; 95% CI, 0.44–2.94). Women who had ≥2 children and had a last birth ≥10 years before recruitment were at reduced risk of BC (OR = 0.44; 95% CI, 0.22–0.90) compared with nulliparous women. When analyses where stratified by age at FFTP, women who had a FFTP at <21 years and had ≥2 children had a 64% lower risk of BC than nulliparous women (OR = 0.46; 95% CI, 0.23–0.93), while women who had a FFTP at ≥21 years and had ≥2 children were also at decreased risk, but the decrease was not statistically significant (Table 4).

**Table 4:** Odds ratios and 95% confidence intervals of breast cancer by combination of reproductive variables.

Reproductive variables	N	Matched^a^	p-value^a^	Multivariate^b^	p-value^b^
**Combination: Parity and breast feeding**					
Nulliparous	48/22	1.00		1.00	
≥1 children, never breastfed	27/14	0.89 (0.39–2.05)	0.78	1.14 (0.44–2.94)	0.79
1 child, ever breastfed	66/45	0.71 (0.37–1.36)	0.31	0.81 (0.39–1.68)	0.57
≥2 children, ever breastfed	147/207	0.32 (0.18–0.57)	**<0.0001**	0.52 (0.27–0.98)	**0.04**
**Combination: Parity and time since last birth**					
Nulliparous	48/22	1.00		1.00	
1 child, <10 years since last birth	52/22	1.28 (0.59–2.79)	0.53	1.79 (0.74–4.35)	0.20
1 child, ≥10 years since last birth	30/30	0.48 (0.23–1.00)	**0.05**	0.47 (0.20–1.12)	0.09
≥2 children, <10 years since last birth	80/100	0.38 (0.20–0.71)	**0.003**	0.66 (0.32–1.35)	0.25
≥2 children, ≥10 years since last birth	69/113	0.28 (0.15–0.52)	**<0.0001**	0.44 (0.22–0.90)	**0.03**
**Combination: Parity and age at FFTP** ^**c**^					
Nulliparous	48/22	1.00		1.00	
1 child, age at FFTP <21 years	14/10	0.81 (0.32–2.07)	0.66	1.06 (0.36–3.14)	0.92
1 child, age at FFTP ≥21 years	68/42	0.78 (0.40–1.54)	0.48	0.86 (0.41–1.84)	0.71
≥2 children, age at FFTP <21 years	69/125	0.26 (0.14–0.48)	**<0.0001**	0.46 (0.23–0.93)	**0.03**
≥2 children, age at FFTP ≥21 years	80/88	0.43 (0.23–0.82)	**0.01**	0.59 (0.29–1.20)	0.15

^a^Conditional logistic regression matched on age, country, city district, and health insurance institution (when appropriate).

^b^Conditional logistic regression matched on age, country, city district, and health insurance institution and adjusted for education level, history of benign breast disease, physical activity, and waist circumference.

^c^FFTP: first full-term pregnancy, median age = 21 years.

### Reproductive factors and breast cancer subtypes

Specific reproductive variables were differently associated with ER+ and ER− tumors (Table 5). In multivariate analyses, the risk of ER+ tumors (*n* = 122) was positively related to older age at FFTP (OR = 1.11; 95% CI, 1.04–1.19 per year), longer time between menarche and FFTP (OR = 1.12; 95% CI, 1.04–1.20 per year), and older age at last pregnancy (OR = 1.10; 95% CI, 1.02–1.19 per year), while it was inversely associated with ever pregnant (OR = 0.35; 95% CI, 0.13–0.96), parity (OR = 0.64; 95% CI, 0.47–0.87 per child), time since last birth (OR = 0.92; 95% CI, 0.85–0.99 per year), and history of breastfeeding (OR = 0.23; 95% CI, 0.09–0.58) (Table 5). Older age at menarche (OR = 0.63; 95% CI, 0.45–0.89 per year) and longer duration of breastfeeding (OR = 0.97; 95% CI, 0.94–1.01 per month) were inversely associated with the risk of ER- cancers (*n* = 48). For TN tumors results were similar to those observed for ER− tumors (Table 5).

**Table 5:** Association of breast cancer and reproductive factors by hormone receptor status.

Reproductive variables	ER+ vs Control (*n* = 122)		ER- vs Control (*n* = 48)		TN vs Control (*n* = 36	
	OR^a^ (95% CI)	p-value^b^	OR^a^ (95%CI)	p-value^b^	OR^a^ (95% CI)	p-value^b^
Age at menarche (years)	1.02 (0.87–1.19)	0.84	0.63 (0.45–0.89)	**0.01**	0.67 (0.46–0.98)	**0.04**
Pregnancy ever	0.35 (0.13–0.96)	**0.04**	1.12 (0.22–5.72)	0.89	0.53 (0.07–3.91)	0.53
Age at FFTP (years)^c^	1.11 (1.04–1.19)	**0.003**	1.03 (0.91–1.17)	0.61	0.97 (0.84–1.13)	0.71
Time between menarche and FFTP (years)^c^	1.12 (1.04–1.20)	**0.002**	1.10 (0.97–1.26)	0.15	1.06 (0.91–1.23)	0.49
Age at last pregnancy (years)^c^	1.10 (1.02–1.19)	**0.01**	1.08 (0.94–1.25)	0.29	1.05 (0.89–1.24)	0.55
Time since last birth (years)^c^	0.92 (0.85–0.99)	**0.02**	0.95 (0.83–1.09)	0.44	0.99 (0.84–1.16)	0.88
Parity^d^	0.64 (0.47–0.87)	**0.005**	0.93 (0.54–1.58)	0.78	0.99 (0.51–1.92)	0.98
Breastfeeding ever^c^	0.23 (0.09–0.58)	**0.002**	1.57 (0.46–5.43)	0.47	0.84 (0.21–3.36)	0.80
Duration of breastfeeding (months)^c^	0.99 (0.98–1.01)	0.48	0.97 (0.93–1.01)	0.13	0.97 (0.92–1.02)	0.20

^a^ORs are given for continuous variables (unit of change).

^b^Conditional logistic regression matched on age, country, city district, and health insurance institution and adjusted for education level, benign breast diseases, physical activity, and waist circumference.

^c^Parous women only. FFTP: first full-term pregnancy.

^d^Parity: number of childbirths.

The association between duration of breastfeeding and BC subtypes was further explored using non-linear models. For ER+ tumors, a significant protective effect of breastfeeding was observed within the first 6 months of breastfeeding (OR = 0.74; 95% CI, 0.60–0.91) while for ER- tumors a protective effect was significant for ≥12 months of breastfeeding (OR = 0,87; 95% CI, 0.76–0.99).

## Discussion

This paper reports the first results on reproductive factors and risk of breast cancer in the PRECAMA study, a multicenter population-based case–control study conducted in young women in Latin American countries. Older age at first full-term pregnancy (FFTP) and longer time between menarche and FFTP were positively associated with risk of BC overall, while older age at menarche, number of childbirths, history of breastfeeding, and duration of breastfeeding were inversely associated with risk. In combined analyses, compared to nulliparous women, the lowest risk was observed among women with 2 or more childbirths and a history of breastfeeding.

In our population, most tumors were ER+ (72%), TN tumors represented 21%, of which more than 90% were basal-like type (20%). Ever being pregnant, younger age at FFTP, shorter time between menarche and FFTP, younger age at last pregnancy, longer time since last birth, parity, and history of breastfeeding were significantly associated with a decreased risk of ER+ tumors, while older age at menarche and longer duration of breastfeeding were inversely associated with risk of ER− and TN tumor.

Few epidemiological studies have investigated the role of reproductive factors on the risk of BC in young, premenopausal women^12^. In particular, there is limited information on the specific reproductive factors associated with TN subtypes, as this phenotype is much less frequent than ER+ tumors^7^. Most of the studies published to date included only Caucasian women or Caucasian and African-American or Asian women^12–16^.

A systematic review on the risk of intrinsic BC tumor subtypes, including 38 studies with pre- and postmenopausal BC cases and controls among Caucasian and Asian women, concluded that most established risk factors reflect risk factors for luminal A (ER+ and/or PR+, HER2−) BC and that some BC risk factors may be differentially associated with other intrinsic tumor subtypes^17^. Among reproductive risk factors, number of childbirths and, to a lesser degree, history of breastfeeding had the strongest protective effect for luminal A BC, while younger age at menarche and older age at FFTP were associated with a greater risk. Older age at menarche and history of breastfeeding were protective factors for TN tumors. Our results are consistent with these findings, although, in this review, data were not stratified by age at diagnosis, so it is not possible to disentangle specific risk factors for BC in young women among the results observed.

More recently, a meta-analysis on reproductive behaviors and risk of developing BC, including 15 studies (10 case–control studies, 3 prospective cohorts, and 2 pooled analyses) among Caucasian, African-American, and Asian women, confirmed some of the previous observations^12^. In the studies providing data on BC among young women, higher number of childbirths and younger age at FFTP were associated with a decreased risk of luminal BC (ER+ or PR+ and HER2−), but not of TN tumors, while duration of breastfeeding was associated with a protective effect in both luminal and TN BC, as observed in the overall analyses^13,14^. Two recent studies focused on the same age range as our study^15,18^. In a study conducted in Seattle, including mostly white women, older age at menarche was protective against TN BC^15^. These results were not confirmed in a later study including a more mixed population with a small proportion of Hispanic (3.7%) women^18^.

In the only cohort study that explored the association of reproductive risk factors and BC subtypes among women aged <40 years of primarily Caucasian descent, older age at menarche (>14 years) was significantly associated with a decreased risk in ER+/PR+ tumors, while in ER−/PR− tumors, results were not significant. The results also suggested a protective effect of increased number of childbirths in ER+/PR+ tumors and an increased risk with increasing number of childbirths in ER−/PR− tumors. Breastfeeding appeared to be slightly protective in both ER+/PR+ and ER−/PR− tumors, but none of these results were statistically significant^8^.

In our study, reproductive risk factors for hormone-dependent tumors (ER+) are consistent with published data. Cross-classification of parity and breastfeeding supports a strong protective effect among women who had ≥2 children and had breastfed compared with nulliparous women. Among ER- tumors and TN tumors, older age at menarche was a protective factor as observed in other studies^15–17,19^ and longer time between menarche and FFTP was marginally positively associated with ER- tumors. Ever pregnant or higher number of childbirths were not related to an increased risk of ER− or TN tumors, neither was age at FFTP. The biological explanation for the effect of pregnancy on BC risk is still unclear. Pregnancy has been shown to initiate cellular differentiation in mammary glands and lower susceptibility to carcinogenesis^20^. This could explain the finding that an older age at menarche and shorter period between menarche and FFTP has a protective effect on BC.

A longer duration of breastfeeding was protective against BC. Breastfeeding has been associated with a reduced risk of overall BC, with a reduction in risk of 4.3% for each 12 months of breastfeeding^21^. A systematic review and meta-analysis concluded that breastfeeding in premenopausal women was related to a reduction in risk of 14%, with a sharper decrease within the first 6 months of accumulated breastfeeding^22^. Considering BC subtypes, consistent results have been observed on the impact of breastfeeding on TN BC while some inconsistency has been observed for hormonal dependent BC. In a meta-analysis including 15 studies (3 prospective cohorts, 10 case-control studies and 2 pooled analyses), ever breastfeeding was associated with a reduced risk of developing both luminal A and TN subtypes^12^; while in a larger meta-analysis, including 27 distinct studies (8 prospective cohorts and 19 case– control studies) ever breastfeeding was inversely associated with ER- and PR- BC as well as TN BC, but no significant association was observed with ER+ PR+ BC overall^23^. In a recent pooled analysis of multiethnic studies in the US including a significant proportion of Hispanic women, longer duration of breastfeeding was associated with a borderline reduced risk of TN BC among women under 50 years of age^19^.

Our results are in line with those results and suggested a sharper decrease after 12 months of accumulated breastfeeding on the risk of ER- and TN BC, while the impact on ER+ tumors would occur within the first 6 months. After pregnancy, an extended period of breastfeeding contributes to the functional ripening of the glandular tissue, and its protective effect appears to be stronger in more aggressive tumors, basal-like TN^24^. Several mechanisms have been suggested, including differentiation of breast epithelium, a lower periodic influence of estrogen/progesterone on breast tissue, and excretion of cells with initial DNA damage from the breast ductal tissue^25^.

Very few studies have focused on Latin American women. In a case-only study conducted among Mexican women, comparing TN with luminal A BC, women with an older age at FFTP were less likely to have TN BC, while women with ≥3 full-term pregnancies were more likely to have TN BC. Breastfeeding for ≥12 months was related to a doubling in the risk of TN BC^26^. However, the high correlation between parity and breastfeeding makes it difficult to interpret the results. In addition, comparing risk factors across BC subtypes cannot provide evidence on risk factors for these specific subtypes, because of the lack of comparison with healthy controls.

The strengths of our study include: (1) the focus on young Latin American women, for whom little is known about the distribution of subtypes of BC and associated risk factors; (2) the use of a standardized methodology to collect data across centers; (3) the inclusion of incident BC cases (time between diagnosis and inclusion in the study was within 1 week) and selection of matched population-based controls; and (4) the centralization of tumor phenotyping through IHC in one laboratory, which ensures uniformity of BC phenotyping and therefore homogeneity in classification. Some limitations need to be mentioned. The sample size of the current analysis is limited, and subgroup analyses suffer from lack of statistical power in particular to explore cross-classification of reproductive variables.

This is the first multicenter breast cancer study in young Latin American women. Our results are in line with previous literature on risk factors for ER+ tumors in other populations. Estrogen receptor-negative and TN tumors were inversely associated with older age at menarche and longer duration of breastfeeding which provide support for breastfeeding promotion among young Latin American women. Given the modifiable nature of breastfeeding and its consistent protective effect on most aggressive tumors, targeted intervention to inform women on its beneficial impact and encourage the practice for period extended at least 1 year should be conducted. As continued accrual of cases and controls expands in our study, we will be able to classify further the subtypes of BC based on molecular markers and confirm these results.

## Data Availability

PRECAMA data and biospecimens are available for investigators who seek to answer important questions on health and disease in the context of research projects that are consistent with the legal and ethical standard practices of IARC/World Health Organization (WHO) and the PRECAMA Centres. The primary responsibility for accessing the data belongs to the PRECAMA centres that provided them. The use of a random sample of anonymised data from the PRECAMA study can be requested by contacting the corresponding author. The request will then be passed to members of the PRECAMA Steering Committee for deliberation.
